# Lectures on the Diagnosis and Treatment of Diseases of the Lungs

**Published:** 1840-12-26

**Authors:** W. W. Gerhard


### MEDICAL EXAMINER.

DEVOTED TO MEDICINE, SURGERY, AND THE COLLATERAL SCIENCES.

No. 52.] PHILADELPHIA, SATURDAY, DECEMBER 26, 1840. [Vol. III.

### LECTURES ON THE DIAGNOSIS AND TREATMENT OF DISEASES OF THE LUNGS.

BY W. W. GERHARD, M. D.

### LECTURE XI.—(*Continued.*)

#### ASTHENIC PNEUMONIA.

Inflammation of the lungs does not necessarily assume the sthenic form; it may be connected with symptoms of depression, either from the beginning, or at an after-period of the disease. In the third stage this naturally occurs to a certain extent; that is, when the suppuration has extended to a considerable portion of the lung, the patient sinks into a prostrate or asthenic condition, very different from the false or apparent prostration which may arise at first from the dyspnœa produced by the extension of the inflammation to a large surface. But the secondary asthenia is not altogether similar in its symptoms to that which occurs much earlier in the disorder, and in its progress differs altogether from it.

The causes which render pneumonia asthenic at the earlier stages of the disorder may be referred to three classes: advanced age, previously enfeebled health, and certain epidemic causes, which are not known. Neglect, and exposure to continued cold, favour the transformation of ordinary pneumonia into this variety, and have some influence over it at the beginning.

The local signs and the expectoration of asthenic pneumonia do not differ from those of the inflammatory variety, except that as it passes more quickly into the suppurative stage there is but little viscid expectoration; it very soon takes on the characters of the third stage, and in some cases the viscid inflammatory sputa are totally absent. There are, however, many exceptions to this rule, and the sputa are sometimes perfectly well characterized.

The general symptoms are more unlike those of ordinary pneumonia: instead of the forcible pulse, and the active excitement of the capillaries, there is a feeble pulse, a diminished action in the smaller vessels, and a rapid sinking of the strength. In the worst cases the prostration is as great as in the typhoid varieties of fever, and the pneumonia is then frequently termed pneumonia typhoides. The epidemics of asthenic pneumonia are often of this character, and the disease is then extremely fatal. Gangrene of the lung frequently supervenes in the third stage of this variety of pneumonia; and in all cases there is a close connection between the two affections, so that it is often extremely difficult to draw the dividing line between them, unless the gangrenous sputa should make the case clear.

The treatment of asthenic pneumonia is a matter of much difficulty; general bleeding is almost never borne with advantage, and in most cases it is directly contra-indicated by the exhaustion of the patient; cupping or leeching is very often of benefit, and in all cases it is easy to try the effects of a small local abstraction of blood, and to abstain from it if its effects should be injurious; in general, this kind of depletion, if borne well, is in such cases of decided benefit. If either the local abstraction of blood should not be tolerated, or the disease should continue, blisters must be applied; they are much more certain in their action than in ordinary pneumonia, and may be used much earlier. The blister often requires to be reapplied if the part should heal very soon, or a new one may be placed over an adjacent part of the thorax. Other contra-irritants, such as sinapisms, are of more benefit as general stimulants to the nervous system, than as revulsives against the pneumonia.

The internal remedies demand more attention, because the proportion of their employment is difficult to find out. Antimony should, as a general rule, be proscribed; but there are some cases in which the inflammatory action is acute enough to justify a recourse to this remedy,—that is, in small doses; in large quantities, it is always of danger. The times for its administration must be carefully chosen. It should never be given if there is much sweating, or a small and feeble pulse. The combination of opium, calomel, and ipecacuanha, is much more frequently prescribed, and, as a general rule, it answers well. The dose may be varied in this form of the disease, just as it is in the advanced stages of ordinary pneumonia; and the opium should be given in minute proportions, not exceeding one grain, or at most a grain and a half in twenty-four hours. In a considerable number of cases I exclude the opium altogether,—that is, if there should be much oppression and difficulty of expectoration.

The stimulating expectorants, and in some cases even wine, or stronger stimulants, are useful, and even necessary, in this disorder. The senega and eupatorium may be given at first nearly in the same doses, as in the third stage of ordinary pneumonia; but they are, in some cases, tolerated for a very short period before it becomes necessary to substitute for them the milder alcoholic preparations, with some nutritious food,—that is, either wine whey, or, in a few extreme cases, milk punch.

In the form of pneumonia which occurs in persons of intemperate habits, and is nearly always asthenic, alcoholic stimulants are often indispensable; this is especially the case if the inflammation should be complicated with delirium tremens. If stimulants should be omitted in this class of individuals, the mortality of the disease will be very great; but if they be combined with local depletion and blistering, the local inflammation will be relieved, while the nervous asthenia, which is so apt to occur in these persons, may be prevented.

Carbonate of ammonia is another remedy which is often of extreme importance in this disorder; it is peculiarly adapted to those cases in which the secretion into the tubes is considerable, and the patient expectorates with difficulty. It may often be combined with small doses of ether, or Hoffman's anodyne. The usual dose is five grains of carbonate of ammonia, and from twenty to fifty drops of the ethereal preparation, every two or three hours; when the depression is very great, the medicine may for a short time be given even in larger doses.

Asthenic pneumonia sometimes prevails as an epidemic, and is attended with so much prostration of strength and alteration of the blood, that it has received the name of typhoid pneumonia, or even of typhus fever. These cases require more decided stimulation than those of the same variety in which the inflammatory symptoms predominate over the general feebleness, and will often scarcely bear even the local abstraction of blood. Blisters, with stimulating expectorants, and sometimes wine, or other alcoholic preparations, become necessary.

### LOBULAR PNEUMONIA, OR PNEUMONIA OF YOUNG CHILDREN.

These terms are used as nearly synonymous, although lobular pneumonia is not strictly confined to children. It is, however, much more frequent in them than in adults. It differs from the ordinary pneumonia both in its progress and pathological conditions. Instead of the disease occurring in one lung, and in a limited portion of the tissue, it is scattered over a large extent, but it attacks isolated lobules, leaving for a time the intermediate tissue in a healthy state; these inflamed lobules becomes more and more numerous, until the whole parenchyma is gradually consolidated. It is this progress of the disease which gives to it the term lobular pneumonia; the lobules affected are chiefly at the posterior part of the lung, for the gravitation of the blood towards this portion favours the development of the disease.

The appearance of the tissue is different from that of ordinary pneumonia; it is much darker, harder, smoother, and imperfectly granulated; it rarely presents the characters of the third stage, passing with difficulty to purulent secretion. The pleura covering the hardened tissue is sometimes, but not always inflamed, and if but few lobules are attacked, there is little or no accompanying pleurisy. The disease is rarely confined to a single lung; both are almost always attacked, but the right lung at an earlier period and to a greater degree than the left. The bronchial tubes are much more frequently inflamed than the pleura; they contain the usual viscid mucus of the bronchitis of children.

The affection of the bronchial tubes is often the first step in the series of diseased actions constituting lobular pneumonia, and the induration of the lungs follows at various periods of time after the commencement of the bronchitis. The induration then appears first at the posterior portion of the lungs, and surrounds the smaller and more numerous tubes; it thence advances gradually towards the anterior part. In other cases the induration of the lung takes place very rapidly, after the impression of cold or some other cause of pulmonary congestion. The difference in the mode of attack naturally establishes two varieties of lobular pneumonia; one is acute and primary, the other more chronic, or at least less acute, and secondary to bronchitis, or to some general disorder of the economy.

In either case the symptoms of the disease are nearly the same. The physical signs are at first merely those of the ordinary bronchitis of children; that is, a sub-crepitant or mucous rhonchus, the percussion remaining at first clear, but gradually becoming dull as the disease advances. The dulness is not confined to one side of the chest, as in ordinary pneumonia, but is nearly equal on both sides, hence it is difficult to draw the line of distinction between the sound and that yielded by a healthy lung. The only way of doing this is to fix in the mind a correct idea of the average sound yielded by the healthy chest in children of the age of the patient, and then to institute the comparison. The dulness does not, in the majority of cases, pass into complete flatness, for there is rarely a perfect consolidation of the parenchyma. The respiration is also in most cases not completely bronchial, for the same reason that the percussion does not often become perfectly flat; but it approaches this character more and more nearly as the disease advances, and sometimes offers it to a very decided degree. Previously to this point, however, it assumes several intermediate changes, becoming gradually harsh and incomplete.

The other signs of this affection do not differ from those of ordinary bronchitis of children; there is in both cases cough, but no expectoration, and the dyspnœa gradually increases as the disease advances from point to point of the lung. There is fever, which is sometimes intense; and the disturbance of the circulation extends to the capillaries, which are much congested, especially those of the face, where the redness is in the early stages of the disease extremely marked, forming circumscribed patches on each cheek. This peculiar colour, with the dilatation of the nostrils caused by the dyspnœa, forms one of the best indications of the disease.

The accidental symptoms are those connected with the abdomen and brain; these are, from their nature, very variable. There is almost always more or less disturbance of the digestive functions; sometimes vomiting, and either diarrhœa or constipation. The very irregularity of these symptoms proves their little importance for the diagnosis, and that they are only of value in the prognosis of the disorder. The cerebral symptoms are more constant; the obstruction to the circulation necessarily produces congestion of the brain, which is shown by decided stupor, which, in bad cases, passes into coma, or even active delirium. Now, if these cerebral symptoms become extremely severe, they may, to a great extent, conceal the pectoral signs; for cerebral disorder produces, as an inevitable consequence, a more or less complete obliteration of the symptoms of other organs, or at least it causes a decided diminution of them.

The diagnosis of this disease is obvious enough from the symptoms which I have described, excepting in one respect. As it arises insensibly during the course of bronchitis, there is no precise dividing line between the two disorders; in practice this is of but little moment, for when the diseases approach nearly, they require a treatment which differs but little. There is also a difficulty with one other disease—that of tubercles in the lung; these begin nearly in the same way as lobular pneumonia, and the local, as well as the strictly physical signs, are very similar. At first they cannot be distinguished; but, after a short period, the softening of the tuberculous matter will render the distinction very clear. The prognosis in this variety of pneumonia is, as a general rule, favourable in its early stages; and, indeed, in all cases where it occurs as an acute disease; but is not from the commencement sufficiently severe to cause extreme dyspnœa. In those cases which are strictly secondary, and succeed to chronic, exhausting diseases, the enfeebled state of the patient's health renders the probability of recovery much less. Under all circumstances the disease is attended with more danger than ordinary acute pneumonia, which is very rarely fatal in children more advanced in age, in whom it often occurs.

The treatment of lobular pneumonia varies according to the manner in which the disorder commences. If it begin as an acute disease, with much oppression, and other evidence of active excitement from the beginning, it may require active treatment,—that is, venesection in a few cases, and very frequently leeching to the chest; these remedies are not, however, in most cases imperatively necessary, but they relieve the patient more rapidly and certainly than any other. Blood-letting, in any form, is to be avoided, if possible, in cases of children; and it is only in those stages of inflammation in which the natural secretory efforts of the system seem to be insufficient for its relief, that it should be resorted to. The external revulsive remedies are, to a certain extent, useful in this form of pneumonia, but are less so than in the same disease as it occurs in adults; hence blisters, and other depletory revulsives, although they do relieve, are rarely of benefit until the advanced stages of the pneumonia, and even then are uncertain. Revulsives that act upon a larger surface, and at the same time are slightly stimulating, are much better, such as large mustard poultices. These should be applied not only to the thorax, but also to the lower extremities, especially to the soles of the feet and ankles. A convenient way of making them is to soak thick pieces of bread in vinegar, and to sprinkle them with mustard. In the declining stage, or in the milder forms of the disorder, a simple onion or garlic poultice is an excellent application.

The natural cure of lobular pneumonia is, like that of bronchitis, by secretion from the bronchial membrane; hence, in mild cases of the disease, nothing more is required than the prevention of injurious influences and the use of a few simple remedies, which may favour the natural tendency to bronchial secretion. These are the wine of ipecacuanha, graduated so as to keep just within the point of exciting nausea, either given alone or with a slightly stimulating expectorant; of these, one of the best and most simple is the domestic syrup of onions, or the lac assafœtidæ. If the mucus becomes very abundant in the bronchial tubes, it will often much relieve the patient to increase the ipecacuanha to a dose sufficient to produce vomiting; there is, however, little difficulty on that score,—for the tendency to vomiting is in these cases so great, that very small doses of ipecacuanha will excite it, or it may occur spontaneously. Vomiting is of course to be avoided if the congestion of the lung should extend over a large portion of the parenchyma.

Tartar emetic may be substituted for ipecacuanha if there is much fever; but it is not, as a general rule, equal to this remedy, nor is it as safe. Still, there is no important objection to it, provided it be given in small doses to produce a secretory, rather than a contra-stimulant effect. The other expectorants to which I have alluded under the head of bronchitis, are often advisable in lobular pneumonia; but the rules for their employment present nothing remarkable.

There is a hygienic precaution, which is essential both in acute and chronic lobular pneumonia: the child should never be allowed to remain long upon its back, nor, if the disease be severe, should it be permitted to sleep more than half an hour at a time. If this be neglected, the congestion of the lungs is greatly favoured, and the disease may prove unexpectedly fatal. The child should be gently carried about, or allowed to sit up in bed, or be simply inclined a little towards one side or the other.

It is evident, therefore, that lobular pneumonia differs chiefly from the ordinary disease in its greater extent, and in its frequently assuming more of the congestive than inflammatory form. But there are many exceptions to this, in which the circulation is excited, and decided depletory means are indicated.

### PNEUMONIA OF THE AGED, AND LATENT PNEUMONIA.

In old age, as in early childhood, pneumonia assumes certain peculiar characters, but in the former case it approaches more nearly to certain stages of ordinary pneumonia. The only important difference is the great tendency of the disease to become latent, that is, to lose the ordinary functional signs of the acute inflammation, and to offer little but the feebleness and prostration which occur in most severe diseases, with little cough and little or no expectoration. Hence, the disease is often scarcely suspected, and in a number of cases it is not recognised unless the obscurity of the general symptoms and the dusky purple tint of the face should lead the physician to explore the chest.

When the disease is not strictly latent, it is never so well marked by the ordinary pectoral symptoms as in more vigorous individuals, and passes rapidly through the first and second stages to suppuration. This peculiarity leaves little room, or at least but a short space of time for antiphlogistic treatment, and obliges us to resort, at a comparatively early period, to the more stimulating remedies which are appropriated to the third stage. At the commencement, however, the antiphlogistic treatment is directly indicated, and may sometimes be pushed with nearly the same vigour as in younger persons; but the period for this is short, and sometimes from the first, hardly discernible.

SECONDARY AND INTERCURRENT PNEUMONIA.

Pneumonia is naturally enough of common occurrence as a sequel to many diseases of the lungs, especially bronchitis and consumption. In the former case the original disease is in a great degree absorbed by the more severe but secondary affection; but in the latter the inflammation will go through its stages, and leaves the tubercles nearly as they were at first. This is, however, not always the case; even if the tubercles are not advanced, their progress is occasionally hastened by the pneumonia, and after an attack cf this kind, we often find that gurgling or crackling is heard when there was merely a slight bronchial respiration previously to the pneumonia. In more advanced cases the pneumonia is not unfrequently the immediate cause of death by invading the portions of the lungs which remained free from tubercles, and were therefore essential for respiration. The inflammation may also form an exciting cause of new tubercles in a portion of the lung of a consumptive, or may give rise to them in one previously free from them, but of a tuberculous predisposition. In this case the gray granulations are found thickly disseminated through the part most inflamed, and are evidently of recent origin. If there be not, however, a strong tendency to this disease, pneumonia has less influence in developing tubercles than pleurisy, notwithstanding there seems to be a more natural connection between the former disease and phthisis.

There is nothing peculiar in the management of these complicated cases, except that they bear a less decided antiphlogistic treatment than pure pneumonia, and mercurials must be used more sparingly. The rules for their management are essentially the same as those which I have already laid down.

